# Exploiting Scanning Surveillance Data to Inform Future Strategies for the Control of Endemic Diseases: The Example of Sheep Scab

**DOI:** 10.3389/fvets.2021.647711

**Published:** 2021-07-16

**Authors:** Eilidh Geddes, Sibylle Mohr, Elizabeth Sian Mitchell, Sara Robertson, Anna M. Brzozowska, Stewart T. G. Burgess, Valentina Busin

**Affiliations:** ^1^School of Veterinary Medicine, University of Glasgow, Glasgow, United Kingdom; ^2^Moredun Research Institute, Pentlands Science Park, Edinburgh, United Kingdom; ^3^Boyd Orr Centre for Population and Ecosystem Health, College of Medical, Veterinary and Life Sciences, University of Glasgow, Glasgow, United Kingdom; ^4^Carmarthen Veterinary Investigation Centre, Animal and Plant Health Agency, Carmarthen, United Kingdom; ^5^Surveillance Intelligence Unit, Animal and Plant Health Agency, Weybridge, United Kingdom

**Keywords:** surveillance, sheep scab, diagnostic data, existing data, disease control initiatives, data analysis, temporal aberration detection algorithm

## Abstract

Scanning surveillance facilitates the monitoring of many endemic diseases of livestock in Great Britain, including sheep scab, an ectoparasitic disease of major welfare and economic burden. There is, however, a drive to improve the cost-effectiveness of animal health surveillance, for example by thoroughly exploiting existing data sources. By analysing the Veterinary Investigation Diagnosis Analysis (VIDA) database, this study aimed to enhance the use of existing scanning surveillance data for sheep scab to identify current trends, highlighting geographical “hotspots” for targeted disease control measures, and identifying a denominator to aid the interpretation of the diagnostic count data. Furthermore, this study collated and assessed the impact of past targeted disease control initiatives using a temporal aberration detection algorithm, the Farrington algorithm, to provide an evidence base towards developing cost-effective disease control strategies. A total of 2,401 positive skin scrapes were recorded from 2003 to 2018. A statistically significant decline in the number of positive skin scrapes diagnosed (*p* < 0.001) occurred across the study period, and significant clustering was observed in Wales, with a maximum of 47 positive scrapes in Ceredigion in 2007. Scheduled ectoparasite tests was also identified as a potential denominator for the interpretation of positive scrapes by stakeholders. Across the study period, 11 national disease control initiatives occurred: four in Wales, three in England, and four in Scotland. The majority (*n* = 8) offered free diagnostic testing while the remainder involved knowledge transfer either combined with free testing or skills training and the introduction of the Sheep Scab (Scotland) Order 2010. The Farrington algorithm raised 20 alarms of which 11 occurred within a period of free testing in Wales and one following the introduction of the Sheep Scab (Scotland) Order 2010. In summary, our analysis of the VIDA database has greatly enhanced our knowledge of sheep scab in Great Britain, firstly by identifying areas for targeted action and secondly by offering a framework to measure the impact of future disease control initiatives. Importantly this framework could be applied to inform future strategies for the control of other endemic diseases.

## Introduction

Endemic diseases, though widely accepted in modern livestock farming, pose a significant challenge to livestock health, welfare, and productivity with often serious consequences for public health and food security (1, 2). However, the increased exploitation of existing data sources for animal health surveillance presents a significant opportunity to monitor populations and to develop new strategies for the control of endemic diseases. Scanning surveillance is the term used in Great Britain (GB) to refer to the laboratory-based monitoring of disease trends from voluntary diagnostic submissions originating from a variety of sources, similar to passive surveillance (3, 4). This represents a cost-effective methodology for monitoring a variety of diseases, particularly endemic diseases. Scanning surveillance in GB is predominantly achieved through the Veterinary Investigation Diagnosis Analysis (VIDA) database, which is a collection of all clinical diagnoses made from submissions to the Animal and Plant Health Agency's (APHA's) Veterinary Investigation Centres (VICs), Scotland's Rural College Veterinary Services' (SRUC VS) Disease Surveillance Centres (DSCs), and partner post-mortem examination providers for livestock and wildlife in GB (5). Increasingly, the potential to further the use of existing surveillance data sources is being recognised (2, 6). As such, GB's surveillance strategies are changing and encouraging the exploitation of existing data to complement the introduction of new data sources and develop a more complete picture of endemic diseases of livestock (1, 2, 4).

Sheep scab is an ectoparasitic disease caused by infestation of the skin/fleece with the mite, *Psoroptes ovis* (7). It is an endemic disease of particular economic importance to the sheep industry, costing an estimated £78–202 million per year (8). *P. ovis* is an obligate ectoparasite which abrades the skin of the sheep and, in the clinical phase of infestation, causes extreme pruritus (9, 10). Prolonged infestations can result in hypoproteinaemia from albumin loss, causing ill-thrift and emaciation (11). In GB various actions, including statutory control programmes, have been implemented to achieve eradication (12), yet at present the national farm-level prevalence is estimated to be around 9% (13, 14). As the picture of sheep scab has previously shown a high regional variation in prevalence (12, 14), areas with a high disease burden need to be identified to better focus efforts and resources for disease control. An important concept for monitoring the true prevalence of a disease also includes knowing the proportion of disease within the population at risk. However, utilising diagnostic datasets from the voluntary submission of samples by farmers seeking a diagnosis through their veterinarian such as the VIDA database often lacks appropriate denominator (animal population) data, which can be a limitation for their interpretation by veterinarians and other stakeholders (15).

Many approaches have been trialled in an attempt to control endemic diseases due to their complexity. For sheep scab, since the removal of the statutory control programme in place until 1992 (12), a number of targeted disease control initiatives have been adopted to improve the awareness and knowledge of the disease and to contribute towards control. These initiatives are normally industry- or government funded, run for a limited period of time, and are working towards a set goal such as increasing awareness, providing education or advice on treatment options (16). However, initiatives are often expensive, time consuming, and difficult to coordinate. Therefore, developing techniques to measure the impact of such initiatives could provide guidance on their use as part of a more sustainable and cost-effective approach to control.

To aid in the evaluation of past targeted disease control initiatives and guide their future use, a temporal aberration detection algorithm (TADA) could be employed. TADAs are a model conventionally used as a bio-surveillance tool to detect outbreaks of pathogens in hospital settings (17). The application of a TADA can identify a statistically significant increase in the number of cases over time, from a baseline period which is free from outbreaks. An alarm is raised when the count exceeds the threshold calculated by the TADA, indicating a potential outbreak (18–20). However, the sensitivity and specificity of the model need to be carefully balanced so not to generate an excessive number of false-positive alarms whilst still reliably identifying true outbreaks. The TADA has the potential to offer a real-time evaluation of disease, making them a very important tool within public health. Now, their application for other purposes is also being increasingly acknowledged, particularly within veterinary medicine (6, 21).

Through analysis of the sheep scab diagnostic data held in the VIDA database, this study aimed to further exploit this existing surveillance data to (i) identify current trends, (ii) highlight geographical “hotspots” suitable for targeted disease control measures and (iii) identify a denominator from the VIDA database itself to contextualise the trends of the diagnostic count data for stakeholders. Finally, this study collated and assessed the impact of past targeted disease control initiatives using a TADA in order to provide an evidence base towards developing cost-effective disease control strategies.

## Materials and Methods

### VIDA Data Collection

The VIDA database records all diagnostic submissions made to the APHA's VICs, SRUC VS's DSCs, and partner post-mortem examination providers for livestock and wildlife in GB. Samples are routinely submitted on a voluntary basis from referring private veterinarians and farmers for diagnostic investigations. The submissions can include one or multiple samples containing a variety of sample material (from whole carcases to blood, milk, or faecal samples). When a diagnosis (or multiple diagnoses) is made by a Veterinary Investigation Officer (VIO), the submission is assigned one (or multiple) VIDA codes. VIDA codes are assigned to submissions where the diagnosis meets pre-determined and defined criteria.

For sheep scab, the VIDA database includes diagnoses made by the APHA or SRUC at VICs and DSCs through a standardised and United Kingdom Accreditation Service (UKAS) accredited skin scrape test to directly identify the *P.ovis* mites from skin scrape samples. Skin scrape samples are taken using a scalpel blade on the outside edge of a lesion site by a private veterinarian and are subsequently examined by laboratory staff at the VIC or DSC. Samples are examined under direct microscopy or using a potassium hydroxide digest if the initial microscopy did not detect any ectoparasites (22). In some cases, sheep scab can also be diagnosed from the identification of mites from other sample types such as wool plucks or hair. If a positive sheep scab diagnosis is reached for at least one sample within a submission (of any sample type), the submission is assigned the diagnostic code “390”. For the purposes of this study all submissions that were assigned the diagnostic code “390” (herein referred to as “positive scrapes”) were extracted from the VIDA database, together with their submission date and a regional geolocator (approximating county-level), from January 1995 to September 2019 inclusive. However, due to incompleteness of the data in early years, the foot-and-mouth disease epidemic in 2001 and the subsequent restocking of livestock in 2002 as a result of the outbreak, only data from January 2003 onwards were included in the analysis.

Since denominators such as total sheep population were not easily accessible for use in this study and would not be continuously available to contextualise the count of positive scrapes, alternative denominators were sought from the VIDA database itself. Therefore, two further datasets were extracted from the VIDA database: *total diagnostic submissions* from ovines and the *scheduled ectoparasite tests* from ovine submissions. The *total diagnostic submissions* dataset represents the count of all diagnostic ovine submissions submitted to the APHA, SRUC VS and partner post-mortem providers. These samples could contain any type of sample material (e.g., carcass, blood, faeces, etc.) from an ovine submission. Where multiple samples (of any type) were included within one submission, this was regarded as a single submission. The *scheduled ectoparasite tests* dataset represents the count of the number of ectoparasite tests for ovine submissions scheduled by the VIO. The tests included: the APHA's test code “TC0081” for an ectoparasite examination and the SRUC VS test codes “MicrSk” for microscopic examination of the skin or hair, “Shscab” for sheep scab examination, and “Skpara” for microscopic examination for lice or mites. Where multiple skin scrapes were scheduled for one submission, this was recorded as one scheduled scrape. Both datasets were extracted as a total count per year for the 16-year study period (2003–2018).

### Sheep Scab Initiatives

To identify and collate the details of all targeted sheep scab control initiatives which took place during the study period across Great Britain (GB), a variety of sources were consulted. Primarily, information regarding the initiatives was retrieved from publicly available sources such as peer-reviewed literature, government and industry reports (23–25). Experts from industry and government were also consulted to capture initiatives where there was insufficient to no information otherwise available. National initiatives, i.e., those which took place in one or more of the three countries in GB, were selected as they were designed to reach a larger portion of the population at risk, featured well-defined start and end dates, and had a higher degree of information available from primary sources. All of the initiatives identified were categorised into a “type” pertaining to the planned actions of the initiative to allow grouping of initiatives. These categories were: “free testing”, where the cost of skin scraping tests was waived or subsidised; “knowledge transfer & skills training”, where education was provided through workshops and training sessions; “knowledge transfer & free testing”, where education was provided, coupled with free skin scraping tests; and “legislation”, where new legislation was introduced beyond the scope of the Sheep Scab Order (1997) which was in place prior to the beginning of the study period.

### Descriptive Data Analysis

All analyses and visualisations, unless otherwise stated, were conducted using the statistical programming language R version 4.0.0 (26). Positive scrape submissions where the regional geolocator was missing (*n* = 91) were excluded from analyses requiring this information.

#### Temporal Analysis

The total number of positive scrapes were grouped by year and country (i.e., England, Scotland, and Wales) to assess the temporal pattern of sheep scab across GB. A Poisson regression was then applied to test the effect of year on the total number of yearly positive scrapes.

The total counts of the two potential denominator datasets were directly compared to the number of positive scrapes for the 16-year study period (2003–2018) to estimate their suitability as denominators. The most appropriate potential denominator dataset for the interpretation of trends by stakeholders was subsequently visualised as counts per year alongside the count of positive scrapes.

#### Spatial Analysis

The positive scrape data were provided with a pre-defined regional geolocator, approximate to county-level, which was used to descriptively assess the spatial distribution of sheep scab across GB. The counts were aggregated by region, (i) firstly per year for the full study period and then (ii) totalled across all years. The aggregated totals were mapped using a shapefile provided by the APHA, including the correct boundaries of the regions defined in the dataset. In addition, the location of the DSCs and VICs were determined and plotted by extracting longitude and latitude from their postcodes using the Office for National Statistics (ONS) Postcode Lookup database (27).

### Aberration Detection

The (original) Farrington algorithm was applied to measure the impact of disease control initiatives on the number of positive scrapes recorded in the VIDA database. As the sheep scab initiatives were specific to each country within GB, a separate time series analysis was performed for each country. The Farrington algorithm, which uses an over-dispersed quasi-Poisson regression-based method for weekly aberration detection was applied to the number of positive scrapes per country, aggregated by week in accordance with the ISO 8601 international standard of time and date (28). This was applied using the “surveillance” package in R version 1.18.0 (18, 20, 29). Note that besides the original Farrington method other algorithms were considered and trialled, among them the improved Farrington (30), CUSUM, and negative binomial method (19). Even so, the original Farrington proved to be a suitable algorithm for the particular challenges of this type of surveillance data, such as adjusting for any unknown past outbreaks, not requiring a long baseline period, and the ability to account for any seasonal effect in the data if present (as sheep scab is well established as a highly seasonal disease) (12, 31). In addition, the original Farrington method has been previously (and successfully) applied to other data extracts from the VIDA database (15).

To determine a baseline period for training the model, weekly aggregates for each country were visualised as time series to ensure the baseline period was free of suspected aberrations or disease control initiatives. The threshold was set at 0.01 level of uncertainty to increase the likelihood of detecting only true aberrations as submissions could have been influenced by a number of further factors beyond disease control initiatives. In addition to this, each data series were decomposed into seasonal, trend, and residual components, visually inspected, and seasonality either confirmed or rejected using a Kruskall Wallis test (*p*-values considered statistically significant if *p* < 0.05) (32, 33).

## Results

### Descriptive Analysis

**Figure 1:**
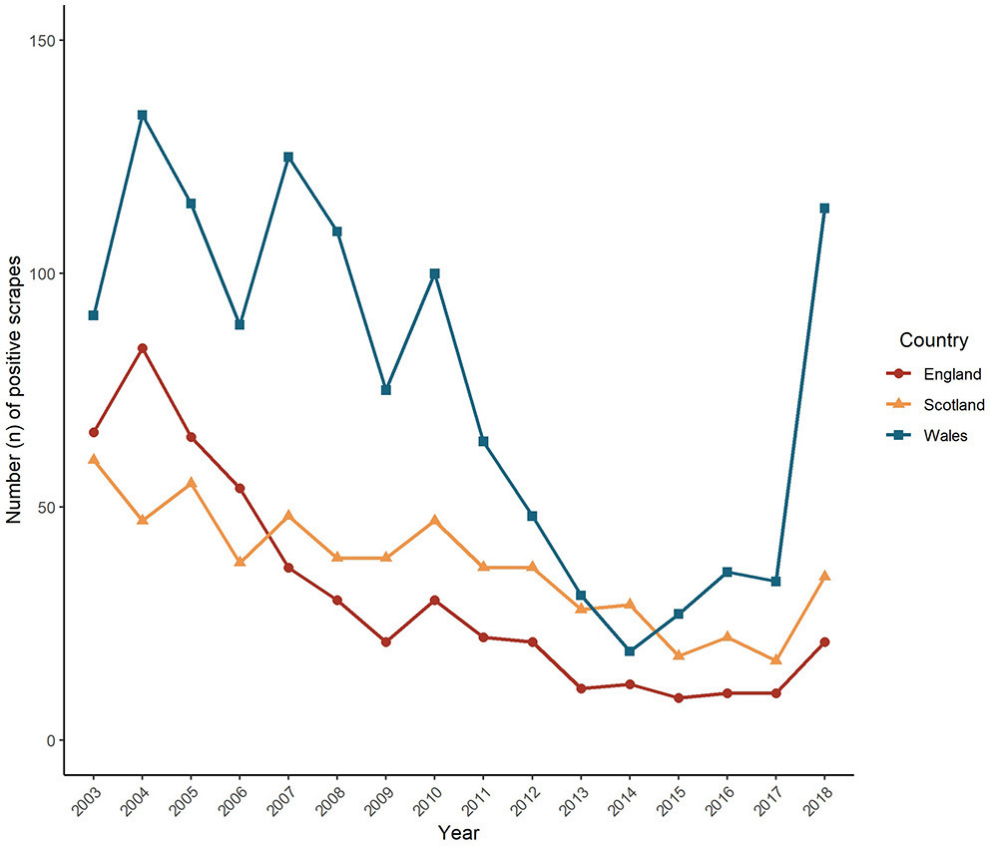
Annual trend of VIDA positive scrapes (sheep scab diagnoses) per country for GB (*n* = 2,310) from 2003 to 2018.

A total of 2,401 positive scrapes were recorded between the 1^st^ January 2003 and 31^st^ December 2018. A significant decrease was observed in the annual count of positive scrapes from the beginning of the study period (*p* < 0.001). The maximum number of positive scrapes was recorded in 2004 (*n* = 277), and the lowest in 2015 (*n* = 55). In contrast to the overall decline observed over the study period, the number of positive scrapes increased by over 2.5 times from 2017 (*n* = 68) to 2018 (*n* = 172). Of the total count of positive scrapes, 2,310 included a geolocator from which the country information could be derived. The annual pattern of positive scrapes per country is displayed in Figure 1. Overall England, Wales and Scotland presented a similar pattern, with a prolonged but fluctuating decline over the study period, with the exception of a sharp increase in counts in Wales in 2018. Wales exhibited consistently higher counts of positive scrapes compared to England and Scotland, with the highest count in 2004 (*n* = 134). The only year where the number of positive scrapes was higher in Scotland (*n* = 29) than in Wales (*n* = 19) was in 2014. In England, the highest count of positive scrapes was also observed in 2004 (*n* = 84), and after a consistent decline, the lowest count occurred in 2015 (*n* = 9). In Scotland, the highest number of positive scrapes was in 2003 (*n* = 60), and the lowest in 2017 (*n* = 17).

**Figure 2:**
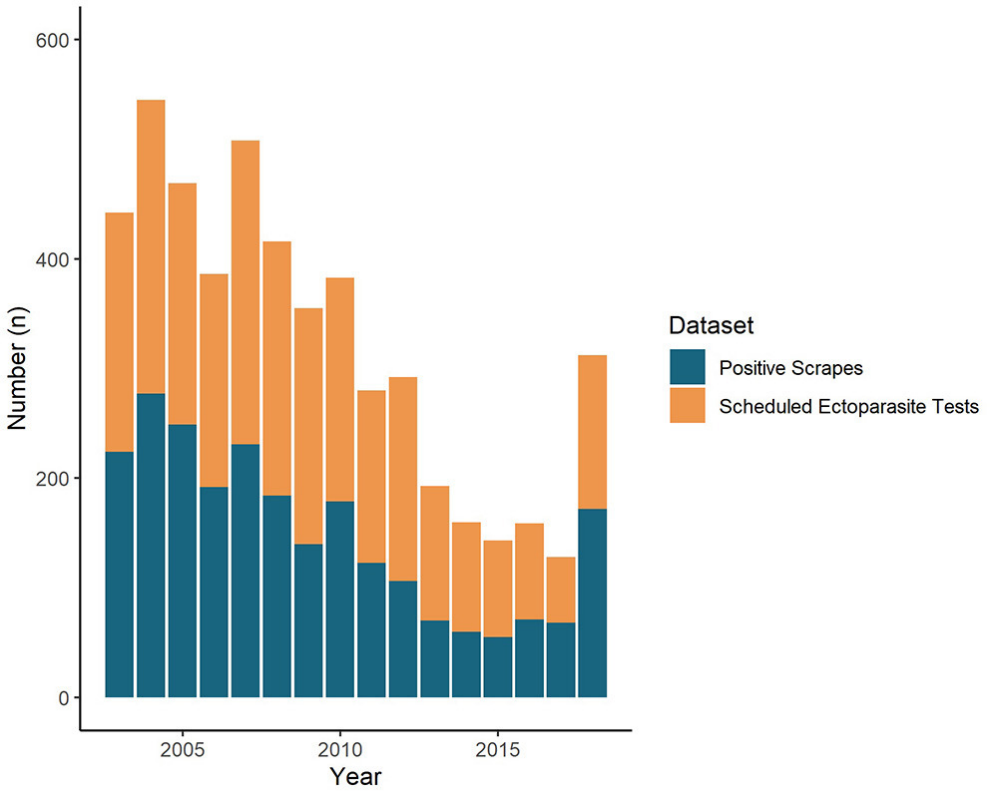
Annual trend of the number of scheduled ectoparasite tests (*n* = 5,171) and VIDA positive scrapes for GB from 2003 to 2018 (*n* = 2,401).

Of the two datasets extracted from the VIDA database as potential denominators, the total *scheduled ectoparasite tests* dataset had a count of 5,171 over the 16-year period. Of this, the count of positive scrapes for sheep scab represented 46.4% of the total *scheduled ectoparasite tests*, and this dataset also exhibited a similar temporal trend to the number of positive scrapes per year, as shown in Figure 2. The total diagnostic submissions dataset had a count of 146,199 submissions, representing a very small proportion (1.6%) of the number of positive scrapes (and as such, was not visualised here).

### Descriptive Spatial Analysis

**Figure 3:**
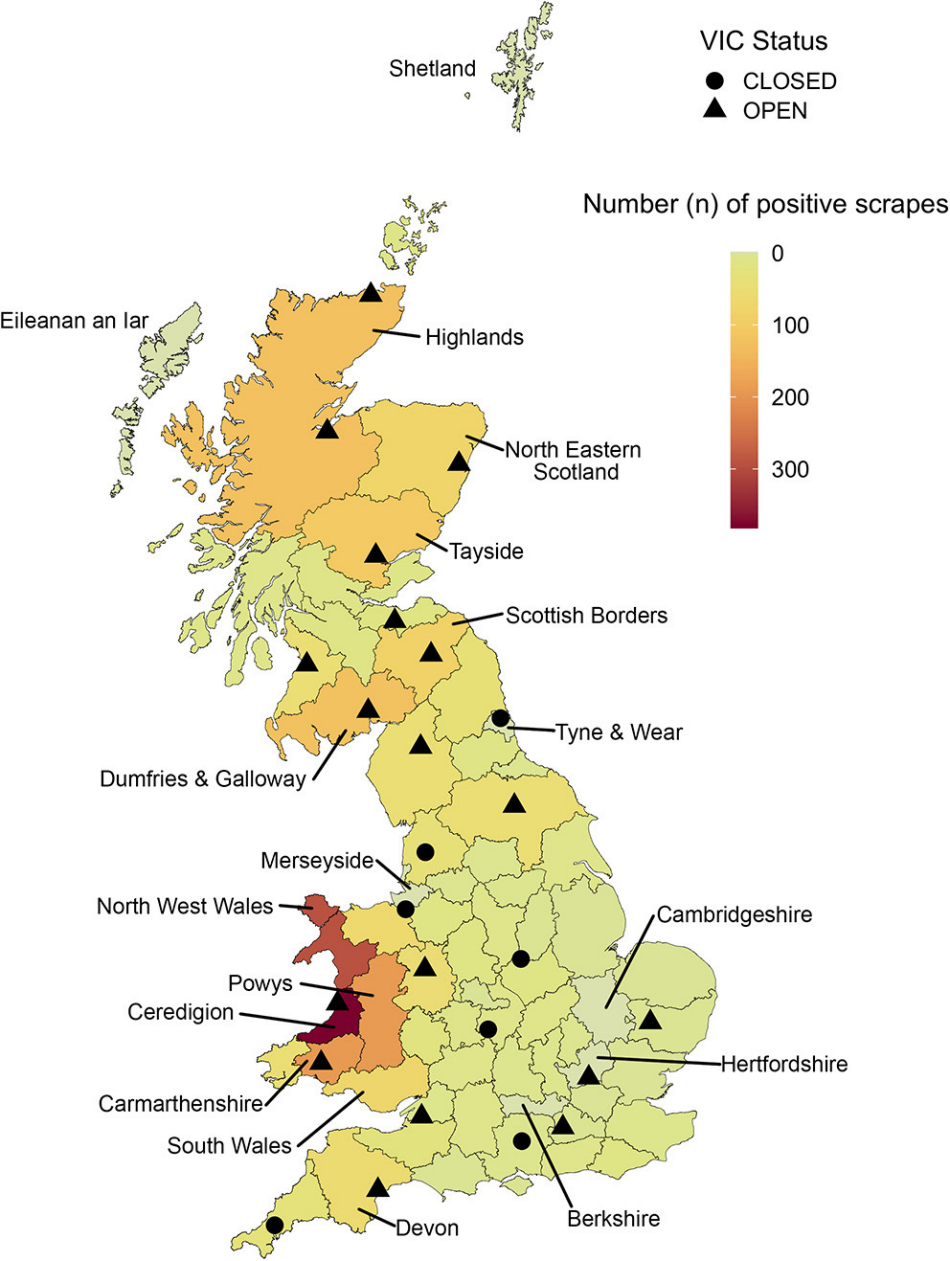
Spatial distribution of the total VIDA positive scrapes (sheep scab diagnoses) from 2003-2018. Points overlaying this represent the DSCs and VICs that were open during the study period. The shape of the point, a circle of triangle, represents the centre's status as of 2020: closed or open, respectively. Labels identify the names of key regions mentioned in text and Table 1.

In total, 2,310 of the 2,401 positive scrapes (96.2%) included a regional geolocator (approximating county-level) which allowed them to be categorised into 69 defined geographical regions across GB (seven in Wales, 14 in Scotland, and 48 in England). At the beginning of the study period, 25 VICs were in operation across GB. As of the end of 2018, 18 were still operational. All closures during the study period took place in England, with one closure in 2013, and the other six in 2014 (Figure 3).

The number of positive scrapes across GB was unevenly distributed, with 52.4% of positive scrapes originating from Wales, 25.8% from Scotland and 21.8% from England. The county with the highest number of positive scrapes across all years was Ceredigion, representing 16.4% of the total diagnoses (Table 1). Ceredigion also represented the focal point within Wales, with the adjacent North West Wales, Powys, and Carmarthenshire also displaying high counts as seen in Table 1. Of the 7 Welsh regions, five were within the 10 regions with the highest total positive scrapes, while the remaining five regions were all in Scotland (Table 1). In England, the region with the most positive scrapes was Devon with 52. Regions with zero positive scrapes within the study period were Berkshire, Cambridgeshire, Hertfordshire, Merseyside, Tyne & Wear, Eileanan an Iar, and Shetland.

**Table 1:** The ten regions with the highest totals of VIDA positive scrapes (sheep scab diagnoses) for GB across 2003–2018.

**Region**	**Country**	**Number of positive scrapes**	**Percentage of the total number of positive scrapes (%)**
Ceredigion	Wales	378	16.4%
North West Wales	Wales	279	12.1%
Carmarthenshire	Wales	189	8.2%
Powys	Wales	188	8.1%
Highlands	Scotland	121	5.2%
Dumfries & Galloway	Scotland	120	5.2%
Tayside	Scotland	103	4.5%
Scottish Borders	Scotland	82	3.5%
North Eastern Scotland	Scotland	75	3.2%
South Wales	Wales	68	2.9%

**Figure 4:**
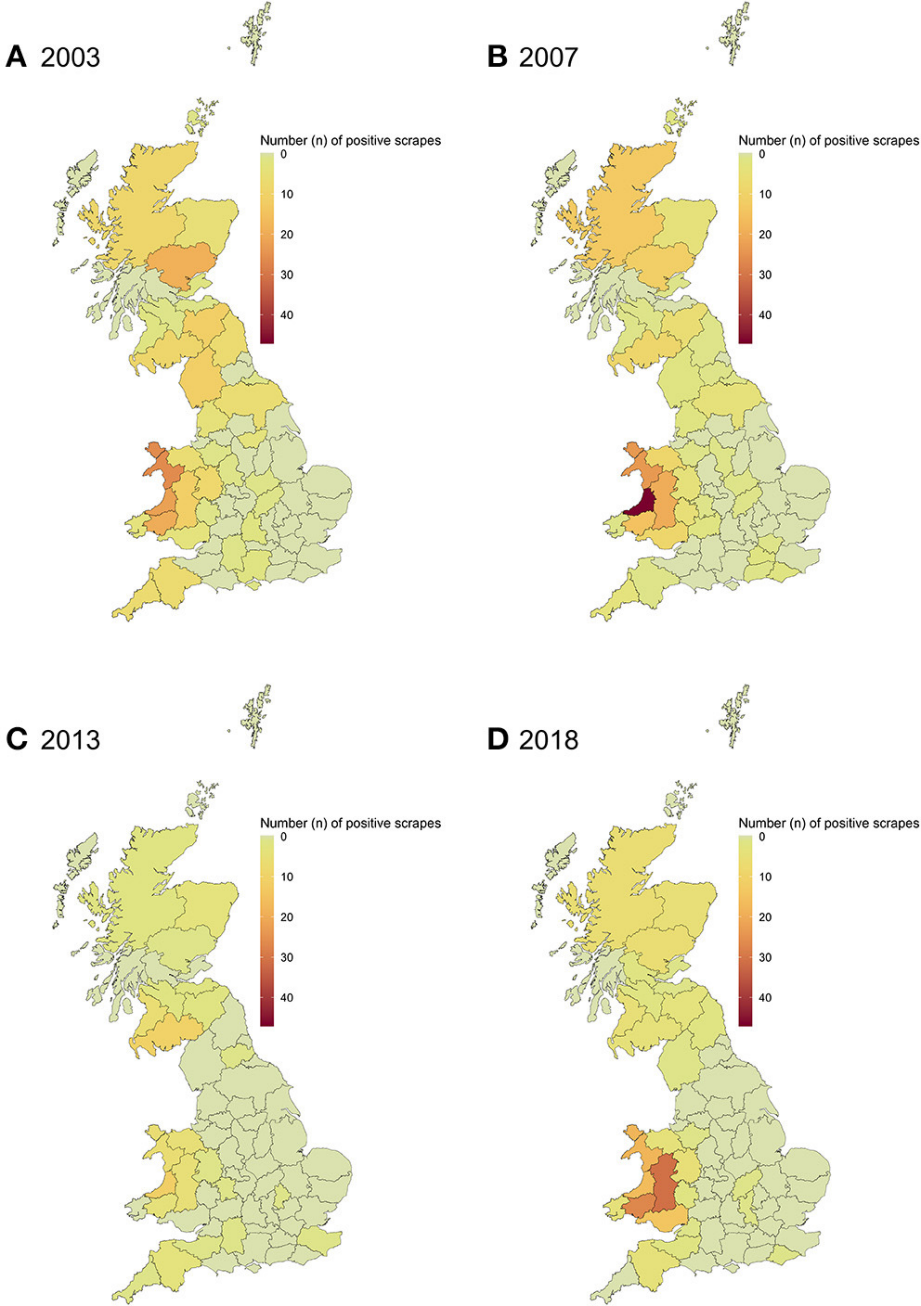
Spatial distribution of VIDA positive scrapes in GB for four key years in the 2003–2018 study period: **(A)** 2003, **(B)** 2007, **(C)** 2013, and **(D)** 2018.

From the study period (i.e. 2003–2018), 4 years (2003, 2007, 2013, and 2018) were selected to represent the spatial distribution of positive scrapes. These years were selected to represent the first and last years of the study period, with the interim years spaced between these years whilst illustrating particular changes in the distribution over time (Figure 4). The count of positive scrapes in 2003 (Figure 4A) saw a maximum of 26 positive scrapes in one region, North West Wales. Overall, the highest number of positives scrapes was seen across the west of Wales, which included Ceredigion, Carmarthenshire and North West Wales (*n* = 20–26) and in Tayside, Scotland (*n* = 11). In 2007 (Figure 4B), Ceredigion observed the highest number of positive scrapes seen in one county across all years, with a total of 47. This peak in Ceredigion also aligned with a more generalised increase in positive scrapes within Wales during 2007 (mean of 17.8 positive scrapes per region). The count in England and Scotland remained low (*n* = <14). In 2013 (Figure 4C), a decrease in the number of positive scrapes occurred across the country, with a maximum of 11 positive scrapes in any region, observed in Ceredigion. In 2018, the low counts (*n* = <7) remained across England and Scotland (Figure 4D); however, counts in Wales varied from 4 in North East Wales to 27 in Carmarthenshire.

### Sheep Scab Initiatives

Within the study period, 11 targeted sheep scab disease control initiatives, as described in Table 2, took place between 2003 and 2018 across GB: 4 in Wales, three in England and four in Scotland.

**Table 2:** Description of the targeted national sheep scab disease control initiatives occurring between the 1st January 2003 and 31st December 2018 in GB.

**Initiative name/organisation**	**Start date**	**End date**	**Initiative type**	**Description**
**Wales**				
APHA^*^	01-12-2003	28-02-2004	Free testing	Period of free skin scrape testing funded and operated by the APHA, operated across England and Wales (*S Mitchell, personal communication)*.
HCC/ Meat Promotion Wales	01-01-2007	28-02-2007	Free testing	Period of free skin scrape testing funded by HCC, an industry-led levy board (*S Mitchell, personal communication)*.
Sheep scab ELISA validation	01-04-2015	01-09-2015	Free testing	Period of free testing to encourage submission of a skin scrape and blood sample to the APHA to validate the sheep scab ELISA. (*S Mitchell, personal communication)*.
APHA	20-12-2017	31-08-2018	Free testing	Period of free testing funded by the Welsh Government and operated by the APHA, after the first reported cases of resistance to macrocyclic lactones were identified (34).
**England**				
APHA^*^	01-12-2003	28-02-2004	Free testing	Period of free skin scrape testing funded and operated by the APHA *(S Mitchell, personal communication)*.
Stamp out Scab	01-01-2013	31-03-2014	Knowledge transfer & skills training	Initiative aimed at knowledge transfer (facilitated by RAMAs for dissemination to clients) and skills training (sessions provided by ADAS veterinarians), instigated by the AHDB and funded through the RDPE (24, 25).
Sheep scab ELISA validation	01-04-2015	01-05-2015	Free testing	Period of free testing initiated by the APHA inviting the submission of a skin scraping and blood sample for the validation of the sheep scab ELISA. (*S Mitchell, personal communication)*.
**Scotland**				
SRUC VS^*^	01-01-2003	10-09-2003	Free testing	Period of free skin scrape testing funded and operated by the SRUC (35).
Scottish Sheep Scab Initiative^*^	11-09-2003	31-12-2006	Knowledge transfer & free testing	A largely industry-led, 3-year long initiative launched at Kelso ram sales initiated by NFU Scotland (36), towards increasing awareness of sheep scab and promoting best practise in disease control through the provision of information (23).
SRUC VS	01-01-2007	16-12-2010	Free testing	Period of free skin scrape testing funded and operated by the SRUC (35).
Sheep scab (Scotland) Order 2010	17-12-2010	Ongoing^†^	Legislation	Mandated the notification of holdings with or suspected to have sheep scab to the local APHA office (37).

* *Initiatives which occurred within the study period but were not included in the analysis*.

† *As of September 2020*.

#### Wales

In Wales, all four initiatives were categorised as “free testing”. The details of the first APHA free testing initiative (operating from 1^st^ December 2003 to 28^th^ February 2004), the Hybu Cig Cymru (HCC)/Meat Promotion Wales and the sheep scab ELISA validation free testing were all similarly sourced from personal correspondence (Table 2). As such, no official report was available on the results of these initiatives. However, a report was available for the second period of APHA free testing (from 20^th^ December 2017 to 31^st^ March 2018) (38) which outlined the intended aims and results of this initiative (Table 2).

#### England

England shared two of its three initiatives with Wales: the APHA free testing (from 1^st^ December 2003 to 28^th^ February 2004), and the sheep scab ELISA validation free testing. The third, instead, was an industry-led “knowledge transfer & skills training” initiative named “Stamp out Scab”, which operated for 15 months and was funded by the Rural Development Programme for England (RDPE). The details of the two initiatives shared with Wales were similarly obtained from personal correspondence (Table 2). Information about the aims and workshops delivered to veterinarians and Registered Animal Medicines Advisors (RAMAs) as part of the “Stamp out Scab” campaign was obtained from the advertising material and previous literature (Table 3).

**Table 3:** Alarms raised by the Farrington algorithm applied to England, Wales and Scotland.

**Country**	**Alarm date**		**Count of positive scrapes**	**Upper threshold**
	**Year**	**Week**		
England	2010	39	4	3.45
Wales	2008	26	4	3.83
	2015	51	5	3.75
	2016	52	5	3.78
	2017	51	8	4.24
	2017	52	9	4.24
	2018	2	12	4.96
	2018	3	16	4.44
	2018	5	7	3.63
	2018	6	4	3.36
	2018	7	5	3.37
	2018	8	6	3.37
	2018	9	5	3.65
	2018	10	4	3.14
	2018	11	4	2.34
	2018	38	3	2.67
*Scotland*	2010	10	3	2.96
	2010	51	6	5.35
	2015	53	3	2.64
	2016	51	5	4.07

*Periods monitored: England 2010-2018; Wales 2007-2018; Scotland 2009-2018. Week is the week number in accordance with the ISO:8601 standard. The upper threshold is the number of counts, as determined by the Farrington algorithm, which would need to be exceeded before an alarm is generated*.

#### Scotland

Uniquely, Scotland offered its initiatives continuously throughout the study period. For the first 8 months SRUC offered free diagnostic testing for sheep scab, similar to the APHA free testing initiatives. Then, the Scottish Sheep Scab Initiative (SSSI) was introduced as a result of industry pressure to control the disease. This was led by industry and government through the Scottish Sheep Scab Industry Working Group, offering advice on best practise coupled with free testing to increase awareness of sheep scab (Table 2). After the SSSI ended, the SRUC free testing resumed and a working group was formed to pave the way towards developing legislation, the Sheep Scab (Scotland) Order 2010. This reintroduced sheep scab as a notifiable disease in Scotland, mandating the reporting of suspected cases (35, 37).

### Aberration Detection

The Farrington algorithm was applied separately for each country due to the devolved nature of animal health in GB, which has been shown to apply to sheep scab through the largely devolved initiatives (Table 2), and differences in counts and trends for each country (Figure 1). Regarding the time series composition, visual inspection of the results suggested that a seasonal effect was present for Wales and Scotland but not for England, which was statistically confirmed through Kruskal-Wallis tests (Wales: *p* = 0.004; Scotland: *p* = 0.007; England: *p* = 0.230) (Supplementary Figure 1). For all countries, the highest number of counts occurred across autumn and winter while counts in the summer months remained low (Supplementary Figure 1).

In Wales, the period of APHA free testing was also excluded from the baseline period, as it was for England. Due to a higher number of counts per week in Wales as opposed to England and Scotland (Supplementary Figure 2) convergence of the model was achieved with a shorter baseline period of 2.5 years, from week 27 of 2004 to the end of 2006. Therefore, the Farrington algorithm was applied across week 1 of 2007 to the end of 2018. This allowed the Farrington algorithm to evaluate three of the four initiatives that occurred across the study period.

**Figure 5:**
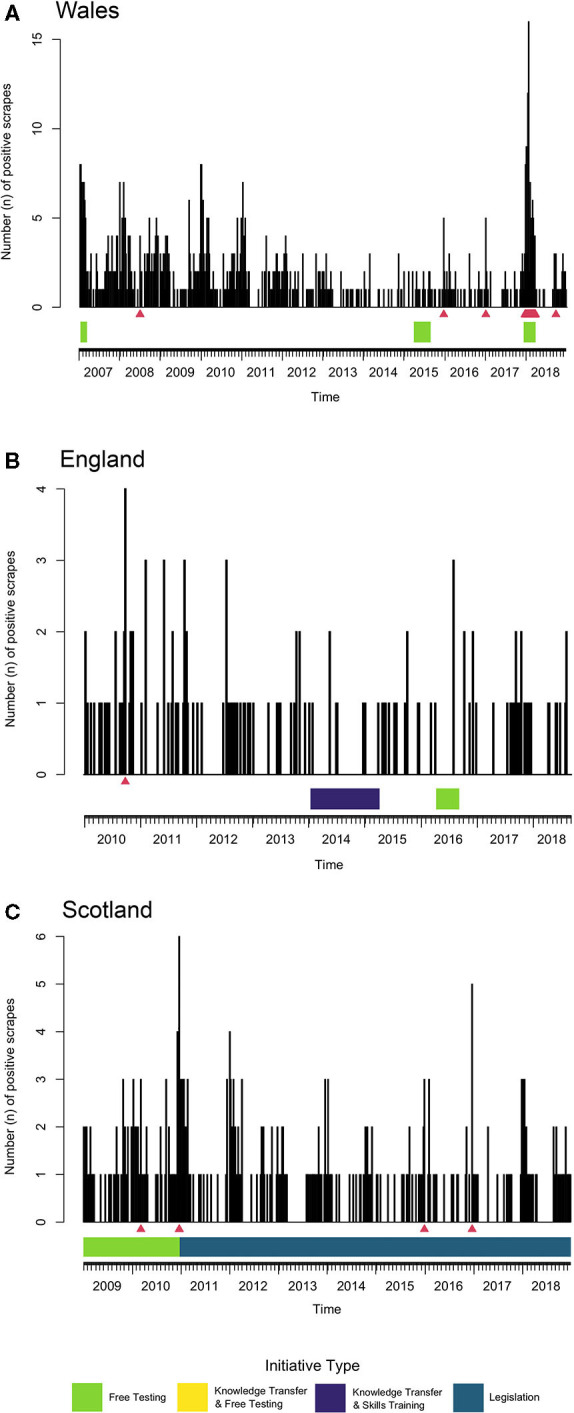
Time-series plot with the Farrington algorithm applied to the count of VIDA positive scrapes in: **(A)** Wales from week 1 of 2007 to the end of 2018, using a reference period of week 27 of 2004 to the end of 2006; **(B)** England from week 1 of 2010 to the end of 2018, using a reference period of week 1 of 2006 to the end of 2009; **(C)** Scotland from the beginning of 2009 to the end of 2018, using a reference period from the beginning of 2005 to the end of 2008. Red triangles indicate alarms raised by the TADA, showing a significant deviation from the expected count. The coloured horizontal bars are a visual representation of targeted sheep scab disease control initiatives in place in that country. These include free testing (green), knowledge transfer & free testing (yellow), knowledge transfer & skills training (purple), and legislation (blue). For description of initiatives see Table 2.

The Farrington algorithm for Wales raised 15 alarms (Figure 5A) from 2017 to 2018. In total, 11 of the 15 alarms (73.3%) occurred from December 2017 to March 2018, falling within the APHA free testing initiative period. The other four alarms did not align with any other known national initiatives. The counts observed on weeks with alarms, compared to the upper threshold produced by the model are displayed in Table 3. The highest number of positive scrapes occurring in 1 week was 16, on the week beginning 15^th^ January 2018. Also, with the exception of two alarms, all alarms occurred in either winter or spring (Table 3).

The baseline period used for England ran from week 1 of 2006 to week 52 of 2009. A later starting reference period was used due to high counts being observed at the beginning of the study period compared with later years (Supplementary Figure 2B), and also taking into consideration the APHA period of free testing from 1st December 2003 to the 28th February 2004 (Figure 5B). Therefore, the study period analysed by the Farrington algorithm was from week 1 of 2010 to week 52 of 2018. The Farrington algorithm raised one alarm during the study period. The alarm was raised in week 39 of 2010 (week beginning 27^th^ September), when 4 positive scrapes were diagnosed, exceeding the upper boundary of 3.45 predicted positive scrapes (Table 3) and also representing the highest count of the weekly time-series for England. This alarm occurred outside the time period of any of the regional initiatives.

Scotland offered initiatives throughout the study period, hence including these in the baseline period was unavoidable. However, the baseline period was adapted to minimise any initial effect from the start of the SSSI. The baseline used was the 4-year period from week 1 of 2005 to the end of 2008, therefore allowing for analysis using the Farrington algorithm from the start of 2009 to the end of 2018 (Supplementary Figure 2C). The Farrington algorithm yielded four alarms, two in 2010, one in 2015 and one in 2016 (Figure 5C). Of the two alarms raised in 2010, the second was raised in week 51, beginning the 20^th^ December, the week after the introduction of the Sheep Scab (Scotland) Order 2010.

## Discussion

As with many endemic diseases in GB, sheep scab will not be eradicated without considerable effort and long-term commitment from all stakeholders, requiring a high level of investment. This is further complicated by the highly variable prevalence of this disease throughout the country. Therefore, the development of targeted, sustainable and cost-effective strategies is paramount to the future success of disease control interventions. In this study, one of the aims was to investigate an existing data source for the scanning surveillance of sheep scab in GB (the VIDA database) to identify current trends and geographical “hotspots” for sheep scab. The analysis confirmed that the spatial distribution of positive scrapes displayed a pattern comparable to previous studies, with high counts observed in Wales, northern Scotland, and northern England. This suggests that prioritising these areas for targeted control strategies could lead to maximum impact. In contrast to previous studies which have found that sheep scab prevalence is either stable or increasing in GB (13, 14, 39), the diagnostic count data analysed here showed a decline in annual counts of positive scrapes for all countries of GB, with the exception of 2018.

Due to the nature of voluntarily submitted diagnostic data, the decline in the count of positive scrapes may reflect a true decrease in sheep scab over the study period, but was likely influenced by many additional factors which need to be considered, including fewer confirmatory diagnoses being sought by veterinarians and farmers. Repeat outbreaks are likely for sheep scab (14), and for flocks where the disease has been diagnosed before, farmers may opt to treat subsequent outbreaks without seeking another confirmatory diagnosis, leading to these outbreaks not being recorded in the VIDA database. A further explanation is that the reduction in submissions for diagnostic sampling may also be influenced by the costs, which currently stand at £24.70 per ectoparasite screen excluding any veterinary costs in England and Wales (40). This is a particular concern for flocks with small profit margins (41, 42). Conversely, in Scotland the submission of ectoparasite screens for cases of suspected sheep scab has been free since 2002 (35). Given that the highest number of positive scrapes originated from Wales and free submissions from Scotland are not substantially higher, it seems unlikely that the decision to submit samples for testing is purely driven by financial factors. It should also be considered that sheep scab is a disease with a large social component due to the associated stigma of having and reporting a sheep scab infestation (which also has negative consequences in Scotland, with movement restrictions being applied). Consequently, there may be little incentive to submit samples, particularly in Scotland. In addition to this, fewer confirmatory diagnostics using the skin scraping methodology may be sought due to (i) veterinary practises using in-house testing, (ii) the closure of some VICs in England and Wales in 2013 and 2014 (43), and (iii) the development and commercialisation of the new diagnostic sheep scab ELISA (44) in early 2017. As the results from in-house testing or diagnostics performed using the sheep scab ELISA are run by commercial companies, they are currently not freely available to support veterinary surveillance.

Somewhat unexpected after the sustained annual decline was the substantial increase in positive scrapes in Wales in 2018, to 3.5 times the counts of the previous year. This substantial increase could raise concern of a true increase in disease prevalence within the country. However, this also corresponded with the APHA free testing initiative in Wales from December 2017 to March 2018 (Table 2), which saw a 500% increase in submissions (38) and likely drives this effect. Although an increase in positive scrapes in 2018 was also seen in England and Scotland, which were not taking part in the initiative, the magnitude was considerably smaller and the effect of the Welsh initiative on disease awareness at national level could not be excluded (38). One of the objectives of our analysis was specifically to identify disease trends and, coupled with the aberration detection analysis of disease control initiatives, identify possible explanations for the apparent increase or decrease in positive scrapes. While a true increase in sheep scab in 2018 cannot be excluded, the free testing initiative has undoubtedly driven the increase in submission and, as a consequence, the number of positive scrapes. To ascertain whether this substantial increase in cases was predominantly due to the offer of free testing, follow up analysis of subsequent years should be carried out.

The “hotspots” (areas with high numbers of confirmed cases) identified in the VIDA data were similar to previous studies, with high counts occurring in Wales and northern Scotland (12, 14). This supports the use of the VIDA database as a suitable means of scanning surveillance, providing a continuous and evidence-based source of information to target areas for disease control initiatives. With further refinement of the geolocators, for example to a county-parish level, the spatial distribution of positive scrapes would allow for more localised control programmes.

As positive scrape submissions recorded in the VIDA database are likely to present only a subset of sheep scab outbreaks, it cannot be used on its own to derive true disease prevalence. Submission might be influenced by factors such as geographical location, awareness of the disease, economic values (of both the disease and the animals), the density of animals in an area, and the number of animals affected (5). To account for the spatial distribution of sheep scab in relation to the sheep population a denominator such as total sheep population from the yearly June agricultural census (45) or density of sheep per holding could be applied to the positive scrapes. These denominators could help highlight additional “hotspot” areas where the sheep population might be small, but many animals are infested. In this study, for example, eight regions (six in England and two in Scotland) had zero positive scrape diagnoses between 2003 and 2018. Some of these areas may be highly industrialised with low density sheep populations, which could explain the lack of sheep scab diagnoses, but in others, it could represent a low presence of disease. As mentioned previously, geographical locations can also have a significant impact on the submission of diagnostic samples. In the case of Eileanan an Iar (the Western Isles off the north west coast of Scotland), since the introduction of the Sheep Scab (Scotland) Order 2010, the Scottish Government reported 32 sheep scab notifications in this region between 2010 and 2019 (46), yet no positive scrapes were recorded in the VIDA database. This suggests that diagnoses have either not been pursued or are made in a different way (e.g., through private veterinarians). It is, however, important to highlight that these are very different datasets; with the Scottish Government notification data recording suspected cases, and the VIDA database representing confirmed positive diagnoses. However, from both databases it is clear that sheep scab is likely vastly underreported in GB, which may be at least in part due to the historic but still present stigma towards the disease among the farming community.

It is important for stakeholders, such as veterinarians, to have an awareness of the overall trends in diagnoses being made when interpreting the positive scrape data to understand external factors which may have influenced the overall submission rate, such as the VIC closures in 2013 and 2014. The total diagnostic submissions dataset offered an insight into the number of diagnostic submissions made across the SRUC VS and the APHA. However, due to the number of other unrelated diagnoses which are included in the VIDA database, positive scrapes represented a very small proportion of this dataset and would not be a very valuable denominator for stakeholders to interpret trends beyond providing supplementary context on how the VIDA database is being used. The *scheduled ectoparasite tests* dataset, which included all diagnostic tests conducted to reach a diagnosis where an ectoparasitic disease is suspected by the submitting veterinarian or VIO, would likely be a more useful denominator obtained from the VIDA database for stakeholders as it could estimate the likelihood of sheep scab from all cases of suspected ectoparasitic disease. This dataset demonstrated that almost half (46%) of the total *scheduled ectoparasite tests* were positive. This highlights the importance of sheep scab in the context of ectoparasitic diseases and demonstrates just how often it is the causative disease when a diagnosis is sought. By analysing the dataset for other VIDA codes, further insight into other ectoparasites (i.e., lice) as differential diagnoses for sheep scab could also be investigated.

The second aim of this study was to investigate the impact of past disease control initiatives and provide recommendations for their future application. The information about the sheep scab control initiatives described here were only available through the organisation(s) that coordinated them, or from personal correspondence. With the exception of results from the APHA free testing from December 2017 to March 2018 being published in a quarterly disease surveillance report (47) and a survey measuring the impact of the SSSI (23), information on the outcome of the majority of initiatives was unavailable. This makes it impossible to determine whether these initiatives were successful without first-hand experience. It was also difficult to locate information pertaining to the operational dates or original objectives of the initiatives as sources were not available publicly. This study has highlighted that there is considerable value in retaining details about these events in the public domain, not only to avoid specific knowledge being only available to the coordinating organisations (and often only to a few people) but also to avoid this knowledge being lost or forgotten. Therefore, to facilitate a more effective approach to information storage about sheep scab control initiatives, it may be beneficial to consider instating a GB-wide database, similar to the USA's centres for Disease Control and Prevention (CDC) list of national health initiatives, which cover a range of diseases important to human health (48). If used prospectively a database could encourage support from other stakeholders, and ultimately offer a more cost-effective alternative by increasing the impact of each individual disease control programme.

The impact of the initiatives was measured using the Farrington algorithm, a TADA commonly used to detect outbreaks of pathogens in healthcare settings (17). Limited previous work has been conducted to investigate the impact of different types of disease control initiatives (23), but the application of the Farrington algorithm could offer a near real-time evaluation. However, the performance of each TADA is highly reliant on the quality of the baseline period supplied. This was very much variable for each country due to conflicts with initiatives and high counts at the beginning of the study period which prevented model convergence, notably for England (Supplementary Figure 2B). In addition, it is possible that aberrations occurred during the baseline which were not known, thus could not be accounted for.

The most common initiatives for targeted sheep scab control were based on free testing and accounted for 8 out of 11 initiatives. The majority of resulting aberrations aligned with the APHA free testing from December 2017 to March 2018 in Wales, which indicates that free testing provoked an increase in diagnostic submissions, achieving one of the main goals of this type of initiative and thus disclosed more disease. Compared to the other types of disease control initiatives shown here, free testing initiatives are much easier to implement and coordinate and, above all, offer a cost-effective way to increase testing at a specific point in time. Yet, more often, only long-term education through knowledge transfer or knowledge exchange can produce lasting changes in mindset and behaviour (49) that could ultimately decrease the incidence of sheep scab. Therefore, there may be potential benefits in combining free testing and knowledge exchange initiatives in future. However, as shown, the impact of knowledge transfer activities is more difficult to quantify. No aberrations specifically aligned with initiatives such as “Stamp out Scab”, a knowledge transfer & skills training initiative. This was likely due to the aim of this initiative not being to directly impact the number of submissions, but to increase the overall awareness of the disease instead. As such, to effectively measure the impact of knowledge transfer initiatives, alternative methods should be sought.

Scotland was in a unique position with initiatives in place throughout the full study period. Therefore, the baseline period had to be set within the SSSI, which likely meant a higher baseline than would have been optimal. Despite this, alarms were still generated: one at the introduction of the new legislation and a further two within the notifiable period suggesting the alarms generated may be representative of true aberrations. Furthermore, this may represent that the notifiable status which was implemented in 2010 has successfully increased the disclosure of sheep scab cases within Scotland.

To summarise, the impact of free testing and legislation initiatives could be measured with the aberration detection analysis as the initiatives caused an increase in positive scrapes. The further use of this method is therefore promising for the application to other endemic diseases and takes into consideration a number of factors including prevalence, awareness, economic burden, and current disease control methods.

In conclusion, the further analysis of an existing scanning surveillance source, the VIDA database, enhanced our knowledge of sheep scab by identifying potential “hotspot” areas for targeted disease control initiatives. It shows a decline in overall submissions, and confirmed that Wales in particular is an area to focus on for future control efforts. Furthermore, scheduled *ectoparasite tests* was proposed as a denominator for stakeholders to interpret the raw number of positive scrapes. Finally, the application of a Farrington algorithm offered a framework to objectively measure the impact of targeted disease control initiatives, something that is being advocated widely as a more cost-effective and sustainable approach to the long-term control of endemic diseases and as a complementary tool in scanning surveillance.

## Data Availability Statement

The data analysed in this study is subject to the following licences/restrictions. Data were obtained from a third party source, the Animal and Plant Health Agency (APHA). Requests to access these datasets should be directed to corresponding author for forwarding to the appropriate contact.

## Author Contributions

EG performed the analysis and wrote the manuscript. VB and SM contributed to the study design, assisted with the analysis, and in writing the manuscript. EM, SR, and AB provided the data for analysis, assisted with the interpretation of the results, and contributed to the manuscript. SB contributed to the study design and contributed to the manuscript. All authors reviewed the final manuscript.

## Conflict of Interest

The authors declare that the research was conducted in the absence of any commercial or financial relationships that could be construed as a potential conflict of interest.
