# Supplementary material for: Exploiting Scanning Surveillance Data to Inform Future Strategies for the Control of Endemic Diseases: The Example of Sheep Scab
Source: Front Vet Sci. 2021 Jul 16;8:647711. doi: 10.3389/fvets.2021.647711 (PMC8322841; doi:10.3389/fvets.2021.647711)
Supplement: Supplementary file 1 [file Data_Sheet_1.pdf]

Supplementary Figure 1A-C: (A) Wales, (B) England, and (C) Scotland.

(A)

(i)

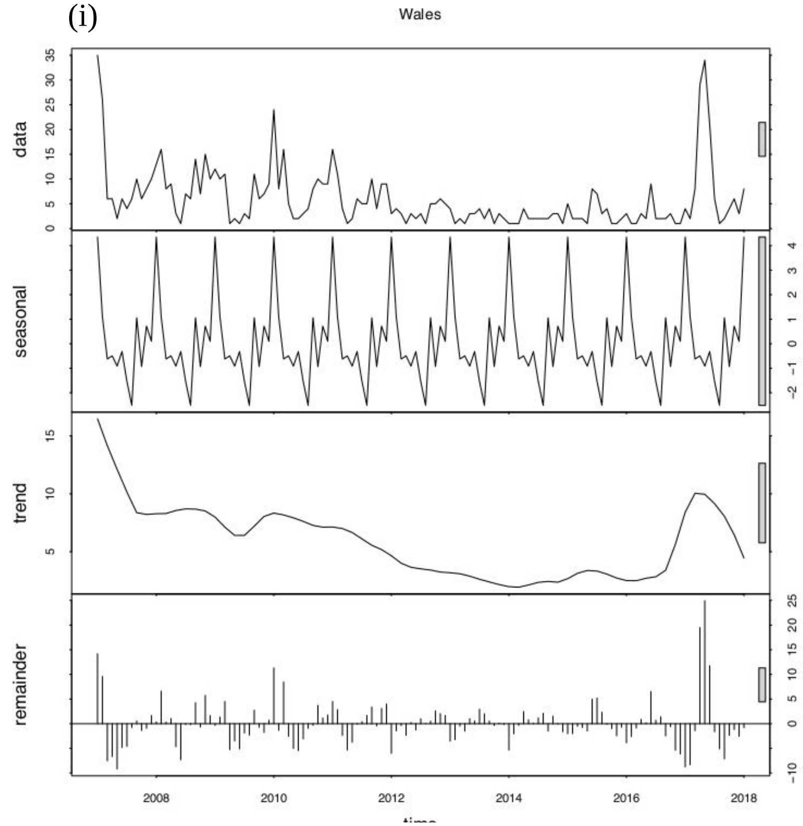

(ii)

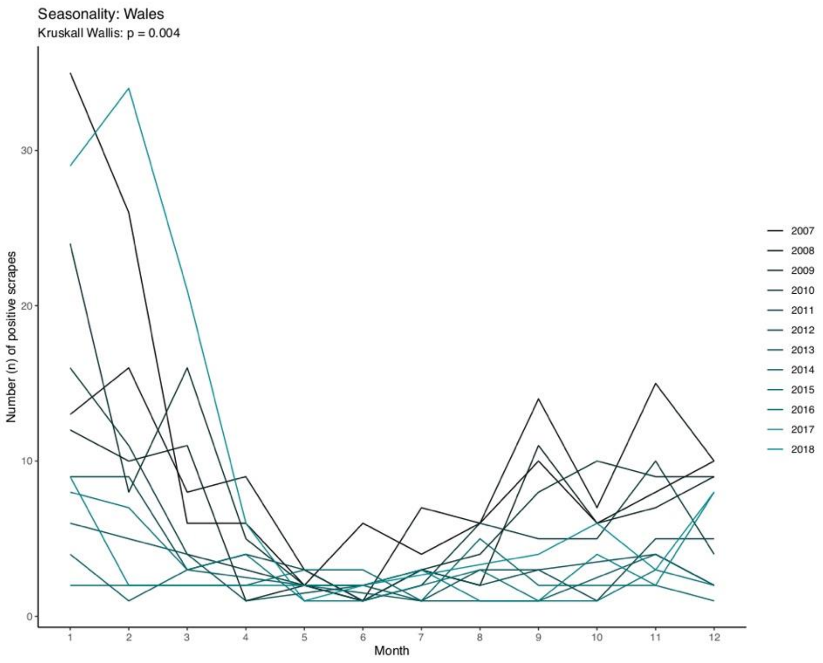

Figure A. (i) Time series of the observed scab submissions for Wales (top) along with the estimated seasonal effects, trend, and random errors. The timeseries decomposition suggests an overall downwards trend (with the exception of Year 2018) and a seasonal effect (measured monthly). Seasonality was confirmed using a Kruskal-Wallis test (p = 0.004). (ii) Seasonal plot showing the underlying seasonal pattern for the Wales timeseries, separate for each year. All years exhibit a similar monthly pattern with higher counts between September and February and low counts in the summer months.

(i) (ii)

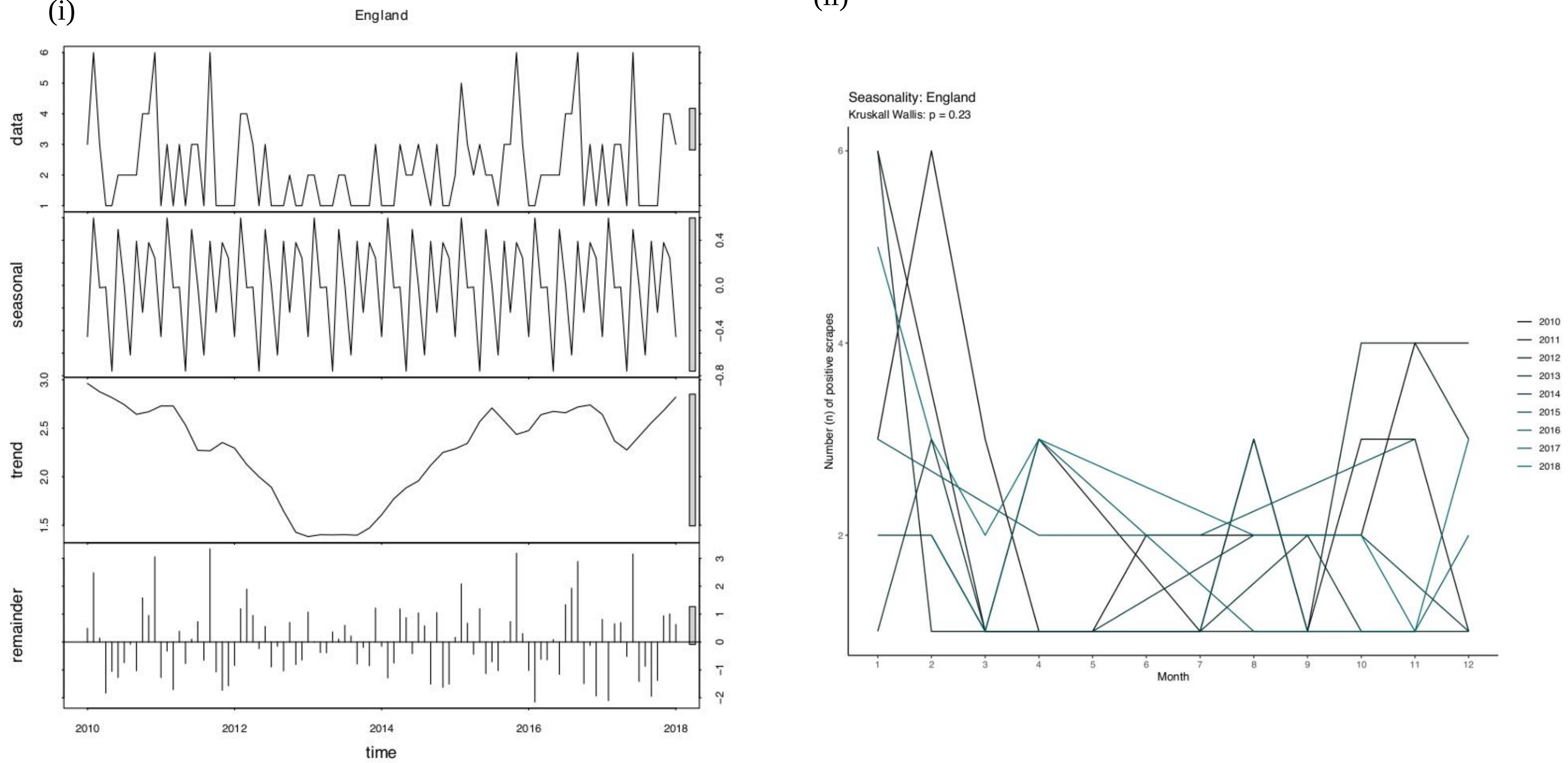

Figure B. (i) Time series of the observed scab submissions for England (top) along with the estimated seasonal effects, trend, and random errors. No seasonality could be established, neither from the time series decomposition nor from the Kruskal-Wallis test (p = 0.230). (ii) Seasonal plot showing the underlying seasonal pattern for the England timeseries, separate for each year.

(C)

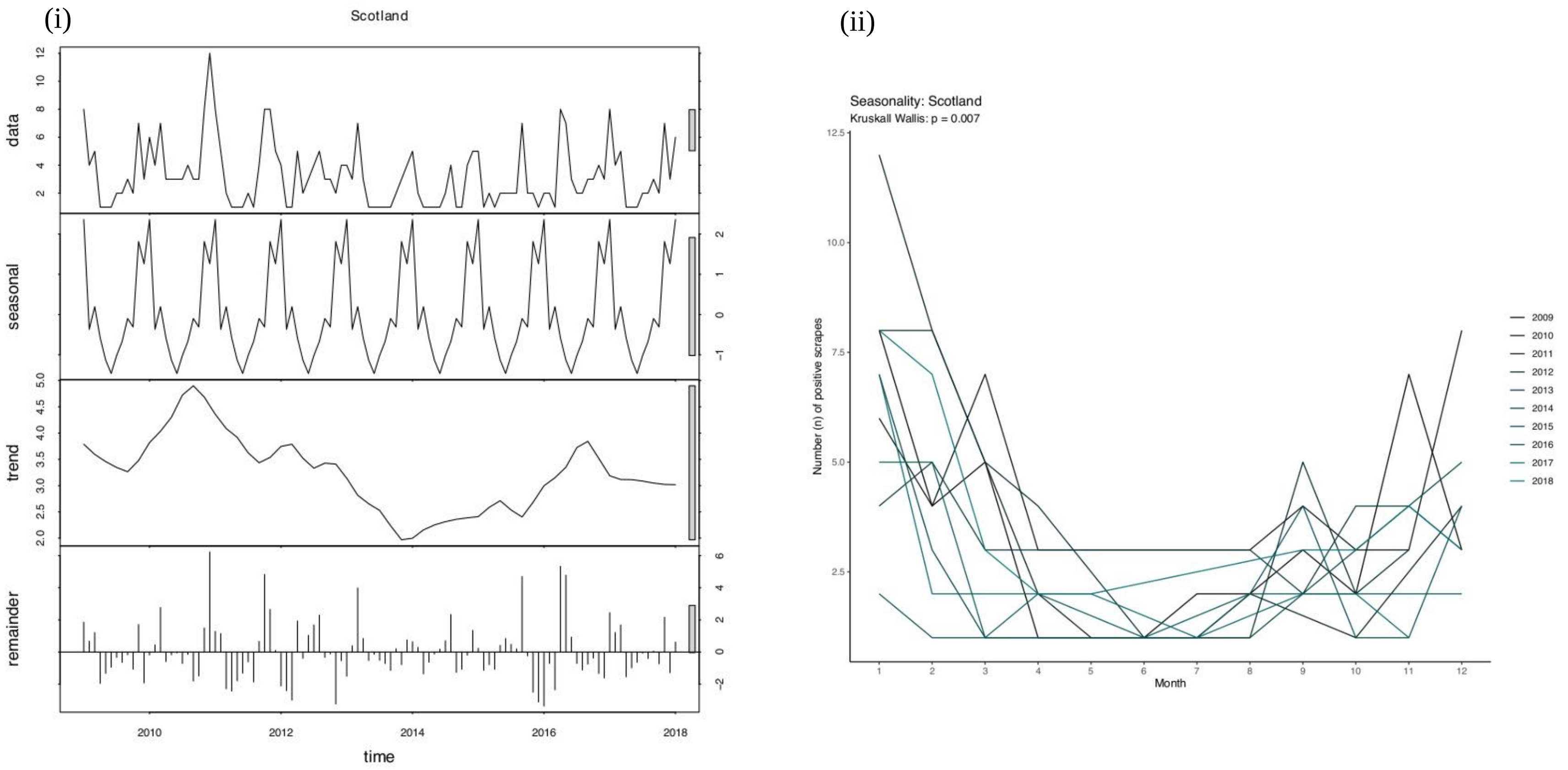

Figure C. (i) Time series of the observed scab submissions for Scotland (top) along with the estimated seasonal effects, trend, and random errors. The timeseries decomposition suggests a seasonal effect (measured monthly). Seasonality was confirmed using a Kruskal-Wallis test (p = 0.007). (ii) Seasonal plot showing the underlying seasonal pattern for the Scotland timeseries, separate for each year. All years exhibit a similar monthly pattern with higher counts between September and February and low counts in the summer months.
