# Supplementary material for: Exploiting Scanning Surveillance Data to Inform Future Strategies for the Control of Endemic Diseases: The Example of Sheep Scab
Source: Front Vet Sci. 2021 Jul 16;8:647711. doi: 10.3389/fvets.2021.647711 (PMC8322841; doi:10.3389/fvets.2021.647711)

**Supplementary Figure 2A-C:** Weekly counts of VIDA positive scrapes (sheep scab diagnoses) for GB from 2003-2018 for: (A) Wales, (B) England, and (C) Scotland.

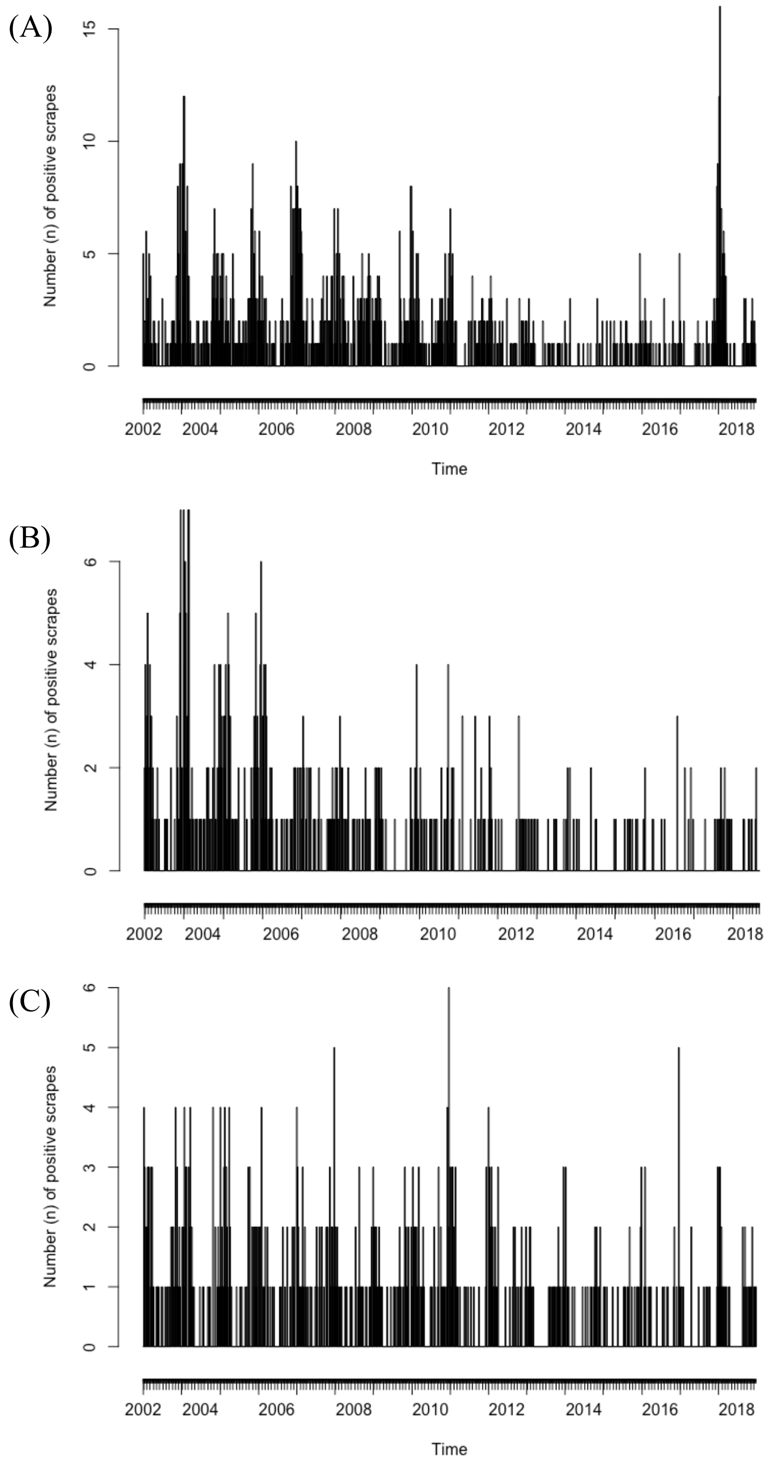

Supplement: Supplementary file 2 [file Data_Sheet_2.pdf]
